# Prescriptions

**Published:** 1897-10

**Authors:** 


A POWDER FOR VAGINAL INJECTION.

The following formula (*Semaine med cale; Progres medical*, July 17, 1897) is attributed to House:

℞. Powdered alum..........
Powdered boric acid.....
Powdered borax........aa 1 ounce
Hydrastine sulphate....9 grains.
Carbolic acid............
Essence of cinnamon.aa 20 drops.

M. For each injection dissolve a teaspoonful of the powder in a pint of water.

INTERCOSTAL NEURALGIA.

According to the *Journal des Practiciens*, Cheron recommends the following prescription for intercostal neuralgia associated with uterine disturbances:

℞. Tincture of gelsemium.100 drops.
Simple syrup..........1½ ounces.
Distilled water...... 6 drachms.

M. Of this two or three teaspoonfuls may be taken twice or thrice a day Such a prescription ought not to be used if the heart is at all feeble.

FOR REMOVAL OF CORNS.

℞. Acid salicylic................ ʒj.

Ext. cannabis indic.......gr. x.

Collodion flex.............. ℥ss.

Sig. To be painted over the corn, twice daily for three days, when an oil poultice is applied over night.

